# Potential Effect of Exosomes Derived from Cancer Stem Cells and MSCs on Progression of DEN-Induced HCC in Rats

**DOI:** 10.1155/2018/8058979

**Published:** 2018-08-27

**Authors:** Faisal A. Alzahrani, Mohammed A. El-Magd, Ahmed Abdelfattah-Hassan, Ayman A. Saleh, Islam M. Saadeldin, Eman S. El-Shetry, Abdelnaser A. Badawy, Saleh Alkarim

**Affiliations:** ^1^Department of Biological Sciences, Rabigh College of Science and Arts, King Abdulaziz University (Jeddah), Rabigh Branch, Rabigh 21911, Saudi Arabia; ^2^Department of Biochemistry, Faculty of Science, King Abdulaziz University, Jeddah, Saudi Arabia; ^3^Department of Anatomy and Embryology, Faculty of Veterinary Medicine, Kafrelsheikh University, Postal Box, Kafr El Sheikh 33516, Egypt; ^4^Department of Anatomy and Embryology, Faculty of Veterinary Medicine, Zagazig University, Postal Box, Zagazig 44519, Egypt; ^5^Department of Animal Wealth Development, Genetics and Genetic Engineering, Faculty of Veterinary Medicine, Zagazig University, Zagazig, Egypt; ^6^Department of Animal Production, College of Food and Agricultural Science, King Saud University, Riyadh 11451, Saudi Arabia; ^7^Department of Physiology, Faculty of Veterinary Medicine, Zagazig University, Zagazig, Egypt; ^8^Department of Human Anatomy and Embryology, Faculty of Medicine, Zagazig University, Zagazig, Egypt; ^9^Department of Medical Biochemistry, Faculty of Medicine, Mansoura University, Mansoura, Egypt; ^10^Department of Biology, College of Science, King Abdulaziz University, Jeddah, Saudi Arabia

## Abstract

Cross talk, mediated by exosomes, between normal stem cells and cancer stem cells (CSCs) in the tumor microenvironment has been given less attention so far. In addition, no publications are available in the literature that address the *in vivo* impact of exosomes derived from CSCs and mesenchymal stem cells (MSCs) on progression of long-term hepatocellular carcinoma (HCC). Herein, we hypothesized that transfer of exosomes among the cells in the HCC microenvironment could either induce or inhibit tumor growth and metastasis depending on their source. To check this hypothesis, we investigated the effect of exosomes coming from two different stem cell populations, hepatic CSCs and bone marrow (BM) MSCs, on progression of long-term DEN-induced HCC in rats and the involved underlying mechanisms. CSCs-exosomes induced a significant increase in liver relative weight and serum levels of cancer markers (AFP and GGT) and liver enzymes (ALT, AST, and ALP), intensive immunostaining for the HCC marker GST-P, and an increased number and area of tumor nodules as compared to HCC rats injected by PBS. CSCs-exosomes also decreased apoptosis (marked by downregulation of *Bax* and *p53* and upregulation of *Bcl2*, and increased immunostaining of PCNA), increased angiogenetic activity (revealed by upregulation of *VEGF*), enhanced metastasis and invasiveness (indicated by upregulation of P13K and ERK proteins and their downstream target *MMP9* and downregulation of *TIMP1*), and induced epithelial mesenchymal transition (marked by increased serum and hepatic level of TGF*β*1 mRNA and protein). Notably, CSCs-exosomes also elevated HCC exosomal microRNA (miR) 21, exosomal long noncoding (lnc) RNA Tuc339, lncHEIH, and the HCC lncHOTAIR and decreased liver miR122 and HCC miRs (miR148a, miR16, and miR125b). All these cellular, functional, and molecular changes were reversed following injection of BM-MSCs-exosomes. However, both CSCs- and MSCs-exosomes failed to change the elevated oxidative stress or the inhibited antioxidant activities induced by HCC. Collectively, our results revealed a tumor stimulatory effect (induction of tumor growth, progression, and metastasis) for exosomes derived from CSCs and an inhibitory effect for exosomes derived from MSCs. These results provide valuable insight on the effect of CSCs- and MSCs-exosomes on HCC growth and progression *in vivo*, which may be helpful to understand the mechanism of HCC development.

## 1. Introduction

Hepatocellular carcinoma (HCC) is a primary malignancy of hepatocyte and the third most frequent cause of cancer-related death globally. Despite notable advances in diagnosis and treatment strategies for HCC in the last decades, the maximum 5-year survival rates remain in patients with advanced HCC due to high metastases and/or postsurgical recurrence rate [1]. The last few decades showed scientific progress in unravelling the signaling molecular pathways in HCC; however, the particular mechanisms regulating HCC are still unclear. Recent studies show that exchange of exosomal contents between different cellular populations in the HCC microenvironment not only could have a key role in tumor initiation and maintenance but also may represent a source of effective noninvasive biomarkers and treatment targets.

Exosomes are extracellular nanovesicles of 30–100 nm diameter that are produced by nearly all cell types [2]. Similar to other exosomes, liver-derived exosomes play an important role in intercellular communication through assuring horizontal transfer of their nucleic acid (i.e., mRNA, micro RNA (miR), long noncoding RNA (lncRNA) molecules, and circulating DNA) and protein cargoes between different cell types in liver (reviewed by [3]). The cellular interaction regulated by these exosomes plays pivotal roles in liver homeostasis [4]. Indeed, liver-derived exosomes induce hepatocyte and cholangiocyte proliferation after injury [5, 6], can activate stellate cells [7], and spread hepatic metabolizing enzymes [8]. In contrast, interaction between HCC exosomal cargo and tumor microenvironment participates in tumor progression, metastasis, and chemotherapy resistance mainly through induction of immunosuppression and angiogenesis [9, 10]. The majority of these exosomal cargoes (i.e., miR18a, miR21, miR221, lncVLDLR, and lncTUC339) are enriched in HCC, with only few miRs (i.e., miR718) exhibiting a notable downregulation in HCC [11, 12]. lncVLDLR mediates the resistance to chemotherapeutic drugs in HCC cells [13, 14], while lncTUC339 and lncHEIH were implicated in onset and progression of HCC [12, 15].

Mesenchymal stem cells (MSCs), as multipotent cells capable of self-renewal and development into multiple lineages, can migrate and interact with tumor cells. Bone marrow-derived MSCs (BM-MSCs) have antitumor activity, induced by interleukin- (IL-) 2 and interferon- (IFN-) b [16]. However, MSC-based therapy associates with many problems, such as the need of a consistent supply of cells, ectopic tissue formation, emboli formation in the pulmonary capillaries, and immunorejection [17]. Furthermore, many studies have indicated that MSCs promote tumor growth, by improving tumor vascularization or creating a tumor stem cell niche [18]. Although the mechanisms by which MSCs regulate tumor cells remain unclear, the interaction between exosomes derived from MSCs and tumor cells is the main player in this effect [19]. Treatment of cancer by exosomes derived from MSCs is more preferable to treatment by MSCs themselves because exosomes are smaller and thus can pass through blood-tissue barriers, and they are less complex than cells and thus can be easily uptaken by cells [20]. Several studies reported that administration of MSC-derived exosomes can relieve the adverse effects of acute liver injury and liver fibrosis [21, 22]. However, to date, only few studies have investigated this effect on HCC. *In vitro*, BM-MSC-derived exosomes induce apoptosis and cell cycle arrest in HepG2 cells [23]. *In vivo*, injection of BM-MSC-derived exosomes inside HepG2-induced subcutaneous tumor in SCID mice suppresses tumor progression [23]. Moreover, intravenous injection of adipose MSC- (AD-MSC-) derived exosomes in an orthotopic model of HCC (induced by rat N1S1-cell inoculation) inhibits tumor progression via upregulation of local and systemic NK cells [24]. miR122 of exosomes derived from AD-MSC increases chemosensitivity of HCC cells both *in vitro* and *in vivo* [10]. Additionally, coadministration of BM-MSCs and tumor-derived exosomes in the presence of IFN-g resulted in decreased proliferation of HCC cells due to cell cycle arrest in the G0/G1 phase [25].

The HCC microenvironment contains a small subset of stem-like cells, cancer stem cells (CSCs), which play an important role in HCC onset, maintenance, and metastasis [26]. These CSCs are likely to originate from malignant transformation of normal residential stem cells (NSCs) in the liver [27]. Thus, the exosomal genetic changes between CSCs and NSCs may happen before those between HCC tissues and normal liver tissues [28]. Exosomes derived from CSCs are important mediators for chemoresistance and tumor metastasis. lncRNA H19 in exosomes derived from CD90^+^ CSCs induces angiogenesis and consequently limits the efficacy of antiangiogenic treatments in HCC [29]. CSC-derived exosomes can also reprogram AD-MSCs into myofibroblast-like cells, which subsequently maintain tumor growth and angiogenesis [30]. This induces MSCs to produce their own exosomes that maintain tumor growth and also alter functions of nontransformed cells in the tumor microenvironment, enhancing their protumor functions (reviewed by [31]). Interestingly, nontransformed cells can also inhibit the proliferation of transformed cells through secretion of exosomes containing antiproliferative miRs and lncRNAs into the tumor microenvironment [9]. However, the aggressive cancer cells usually overcome this inhibitory effect, resulting in tumor progression.

Numerous studies suggest that the exosomes present in the tumor microenvironment play a pivotal role in cancer growth and progression by altering and/or regulating local cellular microenvironments [11, 13, 14, 32]. However, the majority of these studies were performed either on cancer cell lines (*in vitro*) or on short-term animal models of HCC, induced by cancer cell inoculation, which fails to show the therapeutic impact of MSC-derived exosomes on a long-term HCC model. Furthermore, despite the impact of CSC-derived exosomes on HCC progression, to the best of our knowledge, very few publications are available in the literature that address the *in vivo* effect of these exosomes on progression of HCC. Herein, we evaluated the potential effect of exosomes derived from BM-MSCs and hepatic CSCs on progression of diethylnitrosamine- (DEN-) induced HCC in rats and the involved underlying mechanism, with a focus on exosomal miRs and lncRNAs.

## 2. Materials and Methods

### 2.1. Isolation of CSCs from HCC Livers

The procedure followed the previously published protocol [33]. Briefly, collected tumor nodules from the liver of DEN-induced HCC rat were washed, minced into 1 mm^3^ pieces, and then cultured in DMEM medium, supplemented with FBS and 1% penicillin/streptomycin (Lonza, Switzerland). Once a single layer of primary tumor cells was formed (approximately after 3 weeks), cells were harvested with trypsin-EDTA (Lonza) and recultured at 37°C and 7% CO_2_ in a serum-free defined stem cell growth medium (DMEM/F12 medium, containing 2 mM/l L-glutamine, 4 U/l insulin like growth factor 1 (IGF1), B-27 supplement, 15 ng/ml basic fibroblast growth factor (bFGF), and 20 ng/ml epidermal growth factor (EGF) (Sigma-Aldrich)). The majority (70 to 90%) of the cells became adherent, with a few floating cells forming spheres. These spheres were then cultured in DMEM/F12 medium, supplemented with FBS, and the cells became attached and grown into a single-cell layer for 1 week. FBS was removed by a wash with PBS, and defined stem cell growth medium was later added.

### 2.2. Isolation of MSCs from Bone Marrow

Bone marrow-derived MSCs were isolated, according to a previously published protocol [34], by flushing of young male albino rat long bones using sterile PBS. Flushed cells were received in DMEM containing 10% FBS and 1% penicillin-streptomycin-amphotericin B, filtered through a 70 mm filter mesh (BD, Falcon), then centrifuged at 3000*g* for 7 min. Obtained cells were cultured in a 5% CO_2_ incubator at 37°C, and nonadherent cells were washed with frequent medium changing (at 3 h then half medium change every 8 h within the first 3 days, with renewal of the whole medium every week). When cells reached 80–90% confluence (approximately after 2–3 weeks), adherent cells (suspected MSCs) were collected with 0.25% trypsin-EDTA for 2 min at room temperature. Isolated MSCs were used after passage 3.

### 2.3. Identification of Isolated CSCs and MSCs

The stemness of isolated hepatic CSCs and BM-MSCs was confirmed by RT-PCR for genes specific for stem cells, *Nanog* and *Oct-3/4*. The following rat primers were used: *Nanog*, forward 5′-GCCCTGATTCTTCTAGCAAT-3′, and reverse, 5′-AGAACACAGTCCGCATCTT-3′ (amplified 120 bp fragment), and *Oct-3/4*, forward, 5′-CATCTGCCGCTTCGAG-3′, and reverse, 5′-CTCAATGCTAGTCCGCTTTC-3′ (amplified 165 bp fragment). Further confirmation for identification of the isolated CSCs and MSCs was done by flow cytometry (Attune, Applied Biosystems, USA) using specific stem cell markers (five positive markers: anti-CD24, -CD44, -CD133, -EpCam, and -CD90, and two negative markers: anti-CD34 and -CD45), according to the manufacturer's instruction (Becton, Dickinson).

### 2.4. Exosome Isolation and Characterization

MSCs and CSCs were cultured in DMEM without FBS (supplemented with 0.5% of bovine serum albumin); this medium will be collected and called later on conditioned medium (CM). The cell-free CM was collected after 48 h and centrifuged at 3000*g* for 10 min to remove cell debris. The supernatants containing the exosomes were filtered through a 0.42 *μ*m filter and were centrifuged at 12,000*g* for 30 min at 4°C to remove debris and apoptotic bodies. Exosomes were isolated from supernatants by twice ultracentrifugation at 100,000*g* (Optima L-90K; Beckman Coulter) for 90 min/each at 4°C, with an interval wash with PBS [35], and the exosomal protein content was measured by Bradford method. The isolated exosomes were identified by transmission electron microscopy (JEM-2100, Joel Inc.) at 80 kV. The exosomes were pelleted, fixed in 2.5% glutaraldehyde in cacodylate buffer at 20°C for 1 h, and stained with 2% uranyl acetate. The specific structural proteins of exosomes (CD63 (1 : 500), CD81 (1500); Santa Cruz) were verified by Western blotting.

### 2.5. Animals and Experimental Design

Healthy male albino rats (*n* = 80) of similar age (~4 weeks) and weight (~130 gm) were housed in a temperature-controlled (25–27°C) and light-controlled room (12 h light/dark cycle) with free access to food (standard diet) and water. The rats were acclimatized for 14 days before beginning the experimental procedures. All experimental procedures described herein followed the guidelines of the Institutional Animal Care and Use Committee of Kafrelsheikh, Zagazig, and King Abdulaziz Universities and was performed in accordance with the NIH guidelines.

Male rats with matched weights and ages were randomly divided into 4 groups (*n* = 20/group): normal control (Nor), DEN-induced HCC injected by PBS (PBS), HCC-administrated CSC-derived exosomes (CSC-Ex), and HCC treated by MSC-derived exosomes (MSC-Ex) groups. Rats in the normal group (Nor) were administered PBS throughout the experiment duration (22 weeks). The remaining 60 rats were given a single intraperitoneal injection of 200 mg/kg DEN (Sigma-Aldrich) in 1 ml of PBS. Two weeks later, 2-acetylaminofluorene (2-AAF; 150 mg/kg, Sigma-Aldrich) was given orally for 2 weeks to promote the development of DEN-induced hepatic foci. At the 14th week, the left and right lobes of livers were directly injected with 200 *μ*l PBS (PBS group), or either 250 *μ*g MSC-derived exosomes (MSC-Ex group) or 250 *μ*g CSC-derived exosomes (CSC-Ex group) in 200 *μ*l PBS [22]. In the latter two groups, two booster intravenous injections of 250 *μ*g respective exosomes were given at the 16th week and 18th week of the experiment.

At the 22nd week of the experiment, the rats were killed by cervical dislocation under light ether anesthesia, the liver was weighed (absolute liver weight), and then the relative weight of the liver was calculated as a percentage of the absolute liver weight/final body weight.

### 2.6. Evaluation of Serum Biochemical Parameters

Blood samples were collected at the time of sacrifice in serum tubes (vacutainer) and were centrifuged at 3000*g* for 5 min to obtain serum. The serum levels of the liver injury biomarkers (aspartate aminotransferase (AST), alanine aminotransferase (ALT), and alkaline phosphatase (ALP)), liver cancer biomarkers (*α* fetoprotein (AFP) and *γ*-glutamyltransferase (GGT)), and albumin were measured using commercially available kits. The serum level of TGF*β*1 was estimated using rat TGF*β*1 ELISA Kit (ab119558) following the manufacturer's protocol (Abcam).

### 2.7. Evaluation of Liver Lipid Peroxidation and Antioxidant Biomarkers

Liver tissues were homogenized using cold PBS, followed by centrifugation at 5000*g* for 15 min at 4°C. The supernatants were used to measure the concentration of the lipid peroxidation biomarker MDA and the activity of antioxidant enzymes SOD, CAT, and GPX using commercial kits (Biodiagnostics Co., Cairo, Egypt, and Randox Laboratories Ltd., Crumlin, UK) and as previously described [36].

### 2.8. Histopathological Staining

Liver tissue samples fixed in 10% neutral-buffered formalin solution were dehydrated in ethanol, cleared in xylene, and impeded in paraffin to form tissue blocks. The latter were sectioned (4–5 *μ*m), and the slides were either stained by hematoxylin and eosin (H&E) or used for immunostaining. Tumors were graded into four grades: low (grade I), moderate (grade II), high (grade III), and very high (grade IV) according to the Edmondson-Steiner (E-S) criteria [37].

### 2.9. Immunohistochemistry

Liver slides were incubated overnight at 4°C with a polyclonal rabbit anti-rat glutathione S-transferase placental form (GST-P) antibody (1 : 500 dilution, Medical & Biological Laboratories Co., Ltd., Japan) and proliferating cell nuclear antigen (PCNA) antibody (1 : 500, Dako, USA). Bound antibodies were visualized by 3,3′-diaminobenzidine (DAB) substrate kit (Vector Laboratories, USA), and nuclei were counterstained using Mayer's hematoxylin. The number and area (mm^2^) of GST-P-positive foci were analyzed per liver section (cm^2^) as previously described [38], while the PCNA-positive cells (brown color nuclei) were quantified using ImageJ software version 1.44.

### 2.10. Molecular Analysis by Real-Time PCR

Total RNA was isolated from liver tissue (either fresh or frozen) using RNeasy Mini kit (Qiagen, #74104) and as previously described [39]. The cDNA was synthesized by reverse transcription of RNA using Quantiscript reverse transcriptase according to the manufacturer's instructions (Qiagen, #205310). Specific primers for *Bax*, *p53*, *Bcl2*, *TGFβ1*, *NFκB*, *VEGF*, *MMP9*, and *TIMP1* genes were designed by the Primer 3 web-based tool based on the published rat sequence (Table 1). lncTuc339, lncHEIH, and lncHOTAIR were obtained from RiboBio (Guangzhou, China). Quantitative real-time PCR (qPCR) was performed using QuantiTect SYBR Green qPCR Master Mix in a StepOnePlus Real-Time PCR system (Applied Biosystems, USA) and reaction cycles as previously described [40]. The quantities of the critical threshold (Ct) of target genes were normalized with quantities (Ct) of the internal control (*β-actin*) as previously described [41]. Levels were expressed relative to normal control samples.

### 2.11. Quantification of miRs

Total miRs were isolated from liver tissue using TRI Reagent LS and mirVana RNA isolation kit (Sigma-Aldrich) according to the manufacturer's procedure. A volume of 6 *μ*l isolated miRs was reverse transcribed using TaqMan miRNA reverse transcription kit (Applied Biosystems), according to the manufacturer's protocol. The expression profiles of miR21, miR122, miR148a, miR16, and miR125b were measured by qPCR using the TaqMan microRNA assays (Applied Biosystems). The relative sample miR expression was adjusted using miR484, as an internal reference [42], with the equation 2^−∆∆Ct^.

### 2.12. Western Blotting

The liver tissues were lysed in RIPA buffer, and the protein concentration was determined by the Bradford method. Equal amounts of protein were loaded and separated on 10% SDS-PAGE gels. Proteins were transferred to a 0.45 *μ*m polyvinylidene fluoride membrane (Millipore). After incubation with the primary antibodies overnight at 4°C, membranes were incubated with horseradish peroxidase- (HRP-) conjugated goat anti-rabbit antibodies (1 : 5000; Santa Cruz) for 1 h at room temperature. The specific protein bands were developed using tetramethylbenzidine (TMB, Sigma). The densitometry analysis of protein bands was carried out using ImageJ software. The density of each band was normalized by *β*-actin. Sources and dilution factors of primary antibodies were TGF*β* (1 : 150; Bioworld), phosphatidylinositol 3-kinase (PI3K, 1 : 100, Abcam), and ERK (1 : 100, Santa Cruz).

### 2.13. Statistical Analysis

One-way ANOVA using GraphPad Prism 5 (GraphPad Software Inc., La Jolla, CA, USA) was used to determine the difference between the groups. Comparison of means was carried out with Tukey's honestly significant difference (Tukey's HSD) test. Data were presented as mean ± standard error of mean (SEM), and significance was declared at *P* < 0.05.

## 3. Results

### 3.1. Identification of Cultured CSCs and MSCs

**Figure 1:**
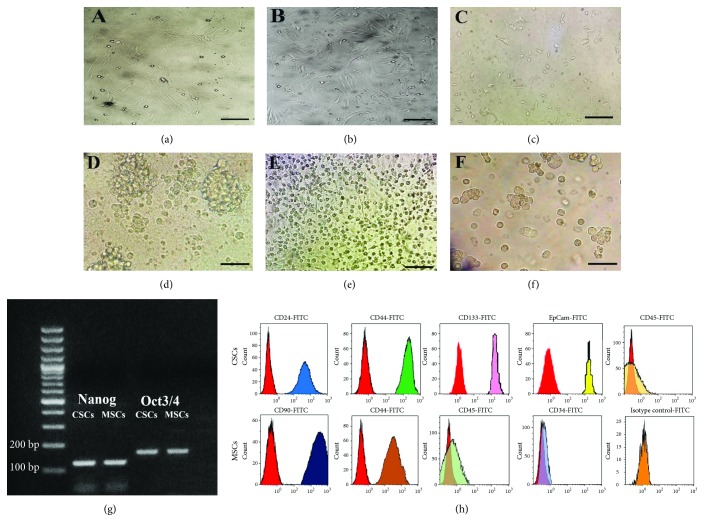
Identification of cultured CSCs and MSCs. MSCs with their characteristic fusiform (fibroblast-like) shape were grown after passage 2 (a) and passage 8 (b). After 2 weeks of plating HCC explants, stem-like cancer cells were grown (c), with characteristic clonal expansion (d) and a very notable proliferative ability (e), and later on few floating cells forming spheres appeared (f). The isolated CSCs and MSCs confirmed by detection of stem cell-specific genes *Nanog* (120 bp) and *Oct3/4* (165 bp) using RT-PCR (g), and by flow cytometry which revealed that CSCs were CD24^+^, CD44^+^, CD133^+^, EpCam^+^, and CD45^−^, while MSCs were CD90^+^, CD44^+^, CD45^−^, and CD34^−^ (h). Scale bars: 35 *μ*m (c–e) and 50 *μ*m (a, b, f).

BM-MSCs were morphologically characterized by their adhesiveness and fusiform (fibroblast-like) shape (Figures 1(a) and 1(b)). Adherent cells were first observed in a culture flask 2 weeks after plating the HCC explants in stem cell-selective media (Figure 1(c)). These stem-like cancer cells were robustly grown with characteristic clonal expansion (Figure 1(d)), and a single layer of cells was formed after 1 week (Figure 1(e)). After culturing the cells in serum-free media, few floating cells forming characteristic spheres of stem cells comprised of 5–10 cells (Figure 1(f)). The stemness of isolated CSCs and BM-MSCs was confirmed by RT-PCR detection of the two stem cell genes *Nanog* (120 bp) and *Oct-3/4* (165 bp, Figure 1(g)). The identity of the cultured cells was further confirmed by flow cytometry. Liver CSCs positively expressed the surface markers CD24, CD44, CD133, and EpCam and negatively expressed CD45, while BM-MSCs positively expressed CD90 and CD44 and negatively expressed CD45 and CD34 (Figure 1(h)).

### 3.2. Characterization and Labeling of CSC- and MSC-Derived Exosomes

**Figure 2:**
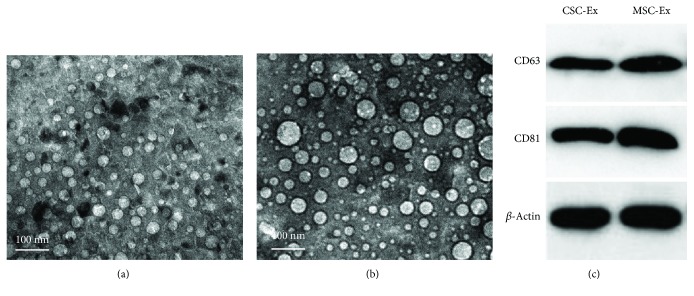
Characterization and labeling of CSC- and MSC-derived exosomes. Transmission electron microscopic examination shows small nanovesicles (30–100 nm) in the sample isolated from the BM-MSCs (a) and hepatic CSCs (b) culture media by ultracentrifugation. Western blot analysis of the culture medium, and exosomes shows presence of CD63 and CD81 in the exosomes of CSC-Ex and MSCs-Ex groups (c). *β*-Actin was used as internal loading control.

Transmission electron microscopy examination showed the presence of nanovesicles with average diameters ranging from 30 to 100 nm in the samples isolated from both BM-MSC (Figure 2(a)) and CSC (Figure 2(b)) media by ultracentrifugation. Western blot results confirmed expression of exosomal specific markers CD63 and CD81 in these exosomes (Figure 2(c)).

### 3.3. Effect of Exosomes on Mortality Rate and Relative Liver Weight

Throughout the experiment, the mortality rate was 10% (2/20), 5% (1/20), and 25% (5/20) in the PBS, MSC-Ex, and CSC-Ex groups, respectively. Moreover, administration of CSC-derived exosomes (CSC-Ex group) in the liver of DEN-induced HCC rats resulted in a significant increase in the relative liver weight (5.52 ± 0.1%, *P* < 0.0001, *n* = 15) compared to that in PBS-injected HCC rats (PBS group, 4.73 ± 0.12%, *P* < 0.0001 versus normal group, *n* = 18) and the normal group (2.95 ± 0.10%, *n* = 20). In contrast, injection of MSC-derived exosomes (MSC-Ex group) led to a significant decrease in the relative liver weights (3.48 ± 0.12%, *P* < 0.0001, *n* = 19) compared with those in the PBS group, but this relative weight remained higher than that of the normal group.

### 3.4. Effect of Exosomes on Biochemical Parameters

**Figure 3:**
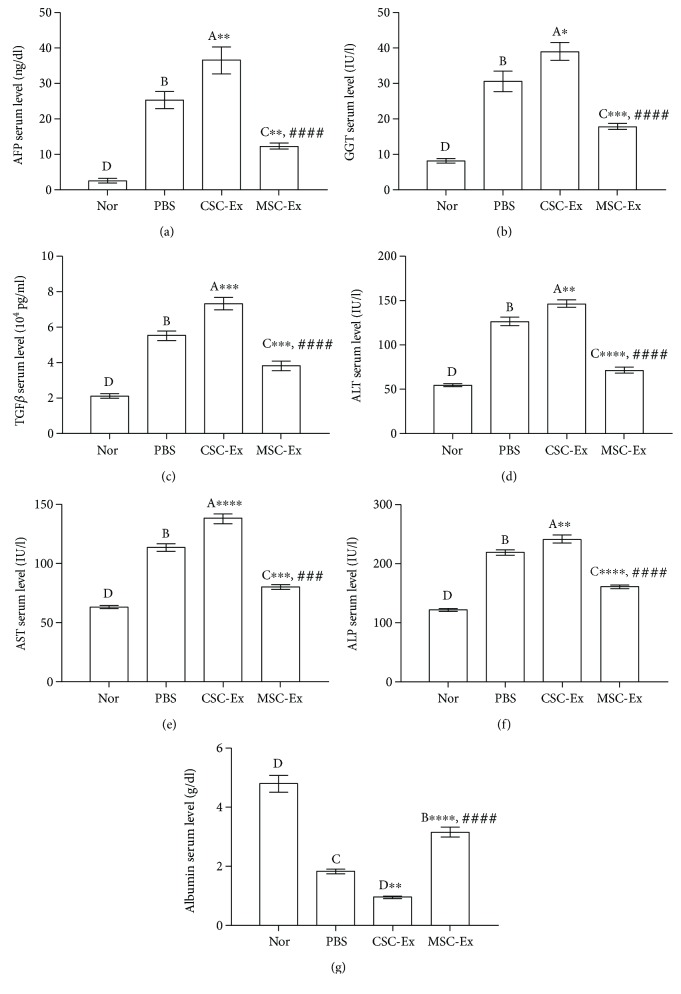
Serum levels of cancer markers (AFP, GGT), TGF*β*, liver damage enzymes (ALT, AST, and ALP), and albumin in normal rats (Nor) and DEN-induced HCC rats treated by PBS or exosomes derived from CSCs (CSC-Ex) or MSCs (MSC-Ex). Values are expressed as mean ± SEM (*n* = 9/group). Different letters on columns mean significant differences. ^∗^*P* < 0.05, ^∗∗^*P* < 0.01, ^∗∗∗^*P* < 0.001, and ^∗∗∗∗^*P* < 0.0001 versus PBS group; ^####^*P* < 0.0001 versus CSC group.

Serum levels of cancer markers (AFP and GGT), TGF*β*1, and liver enzymes (ALT, AST, and ALP) were significantly higher in the CSC-Ex group, followed by the PBS group as compared to the normal group. These elevated levels were reduced in the MSC-Ex group but still slightly higher than that in the normal group (Figure 3). Importantly, serum albumin was significantly higher in the MSC-Ex group than in CSC-Ex and PBS groups.

**Figure 4:**
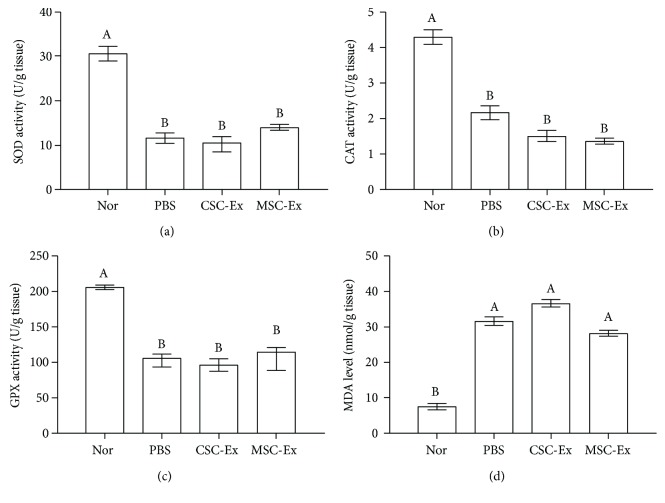
Antioxidant/ROS status of HCC rats after treatment by exosomes derived from CSCs and MSCs showed unchanged levels of MDA, SOD, CAT, and GPX in the liver. Values are expressed as mean ± SEM (*n* = 9/group). Different letters on columns mean significant difference at *P* < 0.0001.

On the other hand, the PBS group showed lower levels of the three antioxidant enzymes (SOD, GPX, and CAT) and higher lipid peroxidation marker MDA levels in liver tissues than those in the normal group. Interestingly, administration of exosomes derived from CSCs and MSCs did not significantly change the levels of these parameters (Figure 4).

### 3.5. Histopathological and Immunostaining Changes Induced by Exosomes

**Figure 5:**
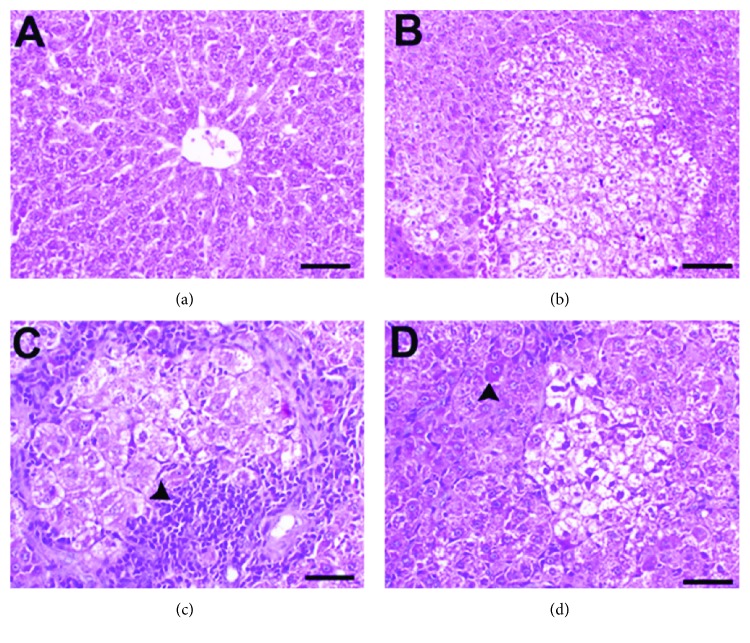
Photomicrographs of H&E-stained liver sections of the normal group (a), the PBS group (b), the CSC-Ex group (c), and the MSC-Ex group (d). Scale bar: a, b, 50 *μ*m; c, d, 30 *μ*m.

Unlike the normal histology seen in the normal group (Figure 5(a)), livers of rats in the PBS group showed altered hepatic foci (AHF) containing large, hexagonal, vacuolated cells (Figure 5(b)). Livers of the CSC-Ex group exhibited numerous proliferative oval cells surrounding AHF (arrowhead, Figure 5(c)). In contrast, the MSC-Ex group had few AHF and oval cells with a notable increase in cellular apoptosis (arrowhead, Figure 5(d)). According to Edmondson-Steiner grades, majority of tumors in the CSC-Ex and PBS groups were of high (grade III) and very high (grade IV) grades, respectively, whereas in the MSC-Ex group all tumors were low (grade I, 10/15 rats) and moderate (grade II, 5/15 rats).

**Figure 6:**
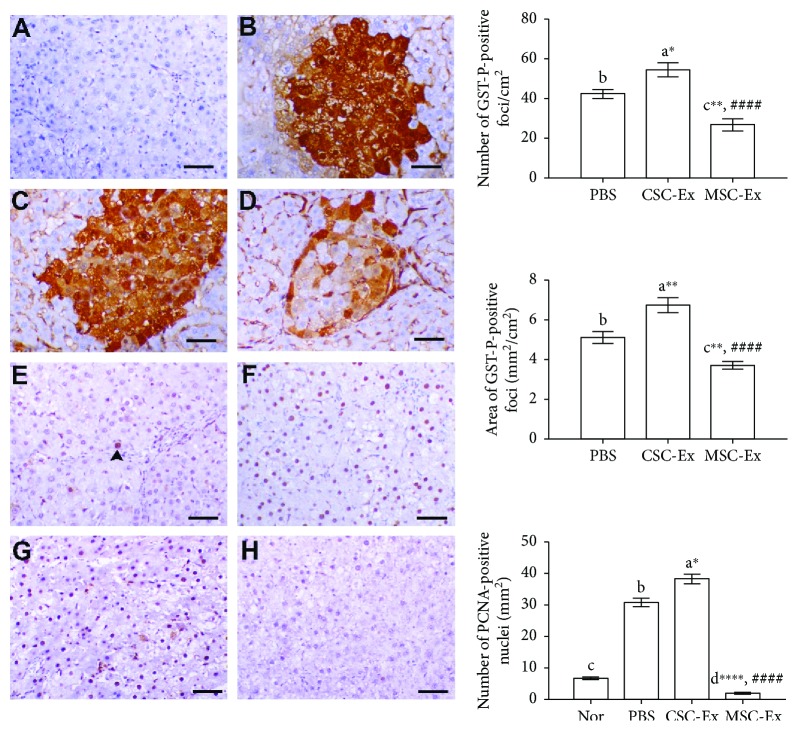
Immunostaining for GST-P (A–D) and PCNA (E–H) in livers of rats in the normal group (A, E), PBS group (B, F), CSC-Ex group (C, G), and MSC-Ex group (D, H). Arrowhead points to PCNA-positive nucleus. Scale bar: (A) E–H, 50 *μ*m, B–D, 30 *μ*m. The graphs show mean number and area of GST-P-positive foci and number of PCNA-positive nuclei in different rat groups. Data was presented as mean ± SEM (*n* = 9). Different letters on columns mean significant difference. ^∗^*P* < 0.05, ^∗∗^*P* < 0.01, and ^∗∗∗∗^*P* < 0.0001 versus PBS group; ^####^*P* < 0.0001 versus CSC group.

Additionally, immunohistochemical examination showed that the mean number and area of the HCC marker glutathione S-transferase placental form (GST-P) positive foci were also significantly higher in CSC-Ex followed by the PBS group than the controls (Figure 6). Again, GST-P-positive foci number and area were significantly reduced in the MSC-Ex group as compared to CSC-Ex and PBS groups. Similarly, the immunostaining index of the nuclear proliferation marker PCNA was significantly lower, mostly within the altered foci, in MSC-Ex as compared to CSC-Ex (highest) and PBS groups (Figure 6).

### 3.6. Molecular Changes in HCC Microenvironment Induced by Exosomes

**Figure 7:**
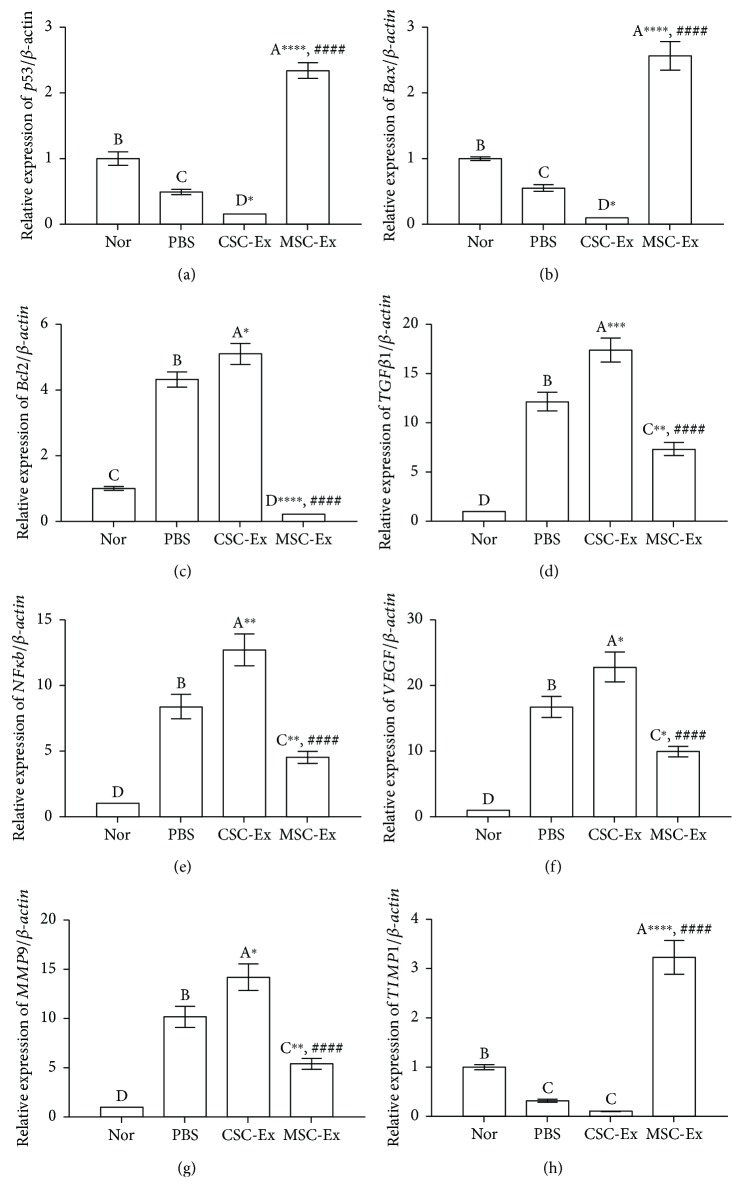
qPCR analysis shows changes in relative gene expression of apoptotic genes (*p53* and *Bax*), antiapoptotic gene (*Bcl2*), EMT-related gene (*TGFβ1*), inflammation-related gene (*NFκB*), angiogenesis-related genes (*VEGF*), and metastasis-related genes (*MMP9* and *TIMP1*) in livers of normal rats (Nor) and DEN-induced HCC rats treated by PBS or exosomes derived from CSCs (CSC-Ex) or MSCs (MSC-Ex). Data presented as fold change from the normal (Nor) control group. Values are expressed as fold change mean ± SEM (*n* = 9). Different letters on columns mean significant difference. ^∗^*P* < 0.05, ^∗∗^*P* < 0.01, ^∗∗∗^*P* < 0.001, and ^∗∗∗∗^*P* < 0.0001 versus PBS group; ^####^*P* < 0.0001 versus CSC group.

To check the underlying molecular mechanism induced by exosomes derived from CSCs and MSCs in HCC, changes in the gene expression of apoptotic markers (*Bax* and *p53*), antiapoptotic marker (*Bcl2*), EMT-related gene (*TGFβ1*), inflammation-related gene (*NFκB*), angiogenesis-related genes (*VEGF*), and metastasis-related genes (*MMP9* and *TIMP1*) were detected in rat livers of all groups and the obtained data is shown in Figure 7. The CSC-Ex group followed by the PBS group showed the most significantly increased and highest *Bcl2, TGFβ1*, *NFκB*, *VEGF*, and *MMP9* mRNA levels and the lowest *p53*, *Bax*, and *TIMP1* mRNA levels. In contrast, MSC-Ex exhibited a significant decrease in the mRNA levels of *Bcl2*, *TGFβ1*, *NFκB*, *VEGF*, and *MMP9* and a significant increase in the mRNA levels of *p53*, *Bax*, and *TIMP1*, compared with the other three groups.

**Figure 8:**
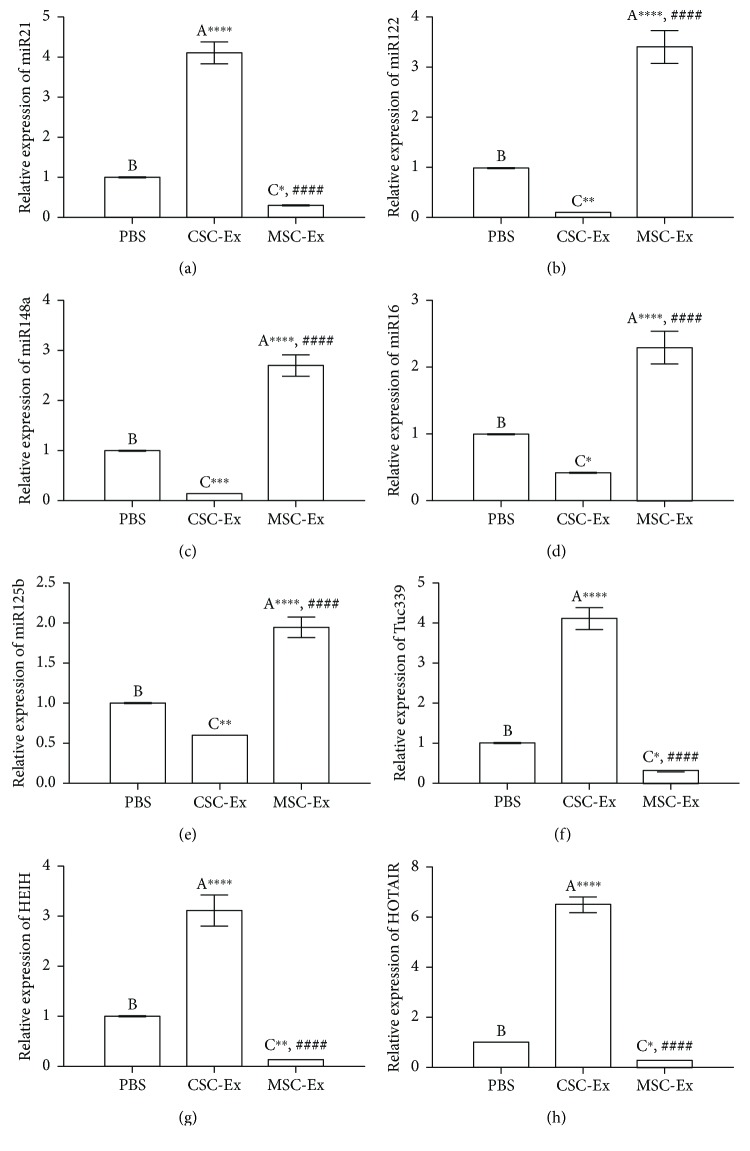
qPCR analysis shows changes in relative gene expression of miR21, miR122, miR148a, miR16, miR125b, lncTuc339, lncHEIH, and lncHOTAIR in livers of DEN-induced HCC rats treated by PBS (PBS) or exosomes derived from CSCs (CSC-Ex) or MSCs (MSC-Ex). Data presented as fold change from the PBS group. Values are expressed as mean ± SEM (*n* = 9). Different letters on columns mean significant difference. ^∗^*P* < 0.05, ^∗∗^*P* < 0.01, ^∗∗∗^*P* < 0.001, and ^∗∗∗∗^*P* < 0.0001 versus PBS group; ^####^*P* < 0.0001 versus CSC group.

To give a deeper insight into the underlying molecular mechanism, we checked the changes in the expression of some miRs and lncRNAs present in the HCC microenvironment. The livers of the CSC-Ex group exhibited a significant upregulation of the exosomal miR21, lncTuc339, lncHEIH, and the HCC lncHOTAIR and a significant downregulation in HCC miR122, miR148a, miR16, and miR125b as compared to the PBS group (Figure 8). In contrast, the livers of the MSC-Ex group showed a significant decrease in the expression of exosomal miR21, lncTuc339, lncHEIH, and the HCC lncHOTAIR and a significant increase in HCC miR122, miR148a, miR16, and miR125b as compared to the PBS and CSC groups.

**Figure 9:**
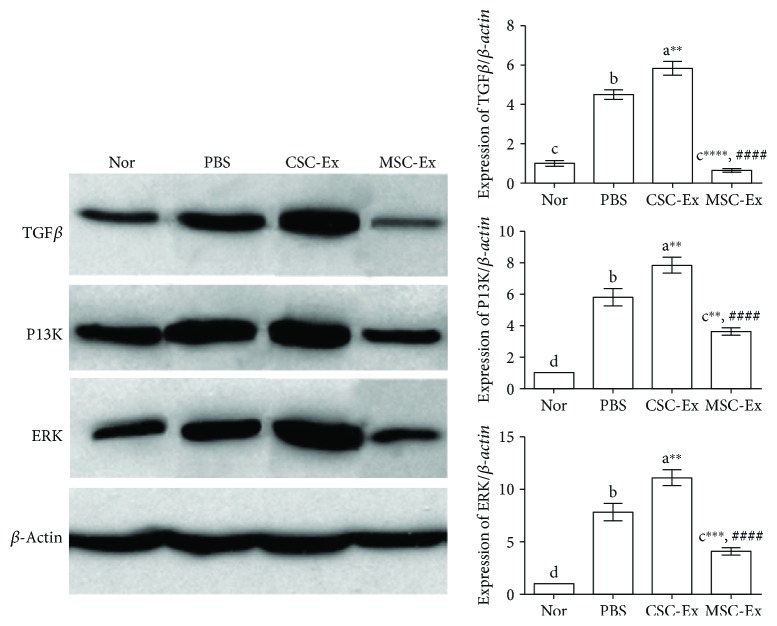
Effect of exosomes derived from CSCs and MSCs on the expression of TGFb, P13K, and ERK proteins in livers of DEN-induced HCC rats using Western blotting. *β*-Actin was used as a control for internal protein loading. Band intensity revealed relative changes in protein expression using the normal group as a control. Values were expressed as mean ± SEM, *n* = 9, from two separate experiments. Different letters on columns mean significant difference. ^∗∗^*P* < 0.01, ^∗∗∗^*P* < 0.001, and ^∗∗∗∗^*P* < 0.0001 versus PBS group; ^####^*P* < 0.0001 versus CSC group.

At protein level, Western blot results revealed a significant increase in the expression of TGF*β*, P13K, and ERK in livers of CSC and PBS groups, with the highest expression in CSC, as compared to the normal group (Figure 9). However, administration of MSCS-derived exosomes resulted in a significant decrease in the expression of these proteins.

## 4. Discussion

Exosomes play an important role in cancer initiation, progression, and metastasis by modulating molecular signaling pathways in tumor microenvironments. Recently, exosome-based therapy has been evolved as a potential inhibitor for HCC progression *in vitro* (HCC cell lines) and *in vivo* (HCC cell lines xenografts in liver or subcutaneous) with minimal complications [23, 24]; however, the efficacy of these exosomes within the hostile hepatic tissue remains a serious concern. HCC exosomes could either induce or inhibit tumor growth and metastasis depending on their source. Indeed, our results collectively revealed a tumor stimulatory effect for CSCs-exosomes and an inhibitory effect for MSCs-exosomes on DEN-induced HCC in rats.

One of the most important issues that render the treatment of tumor ineffective is the presence of CSCs that carry high metastatic, recurrent, and chemoresistant properties [43]. Therefore, it is crucial to target these cells, or their exosomes, for more efficient eradication of tumor. To the best of our knowledge this may be the first study to investigate the potential effect of CSCs-exosomes on HCC progression and metastasis *in vivo*. Expectedly, our results revealed that CSCs-exosomes induced tumor growth, progression, and metastasis. They induced a significant increase in liver relative weight and serum levels of cancer markers (AFP and GGT) and liver enzymes (ALT, AST, and ALP), as well as intensive immunostaining for the HCC marker GST-P, and increased number and area of tumor AHF as compared to HCC rats injected by PBS. Notably, serum albumin was significantly lower in the CSC-Ex group than in the PBS group. This may suggest a negative correlation between albumin levels and HCC progression. In agreement with this notion, it has been investigated that the elevated serum albumin not only is associated with low recurrence rate of HCC in patients but also can inhibit tumor cell proliferation [44].

To further determine the mechanism responsible for the effect of CSCs-exosomes on tumor progression, we detected the expression of some genes known to be important to tumor growth (*Bax*, *p53*, *Bcl2*), metastasis, and angiogenesis (*TGFβ1*, *NFκB*, *MMP9*, *TIMP1*, and *VEGF*) by qPCR. We also evaluated the expression of TGF*β*, P13K, and ERK proteins by Western blotting, quantified immunostaining of the nuclear proliferative marker PCNA by immunohistochemistry, and measured the serum level of TGF*β* by ELISA. Our results confirmed that CSCs-exosomes increased the expression of *Bcl2*, *TGFβ1*, *NFκB*, *MMP9*, and *VEGF* genes; the expression of TGF*β*1, P13K, and ERK proteins; the immunostaining of PCNA; and the serum level of TGF*β*1, but decreased *Bax*, *p53*, and *TIMP1* mRNA levels in livers of HCC rats as compared to HCC rats injected by PBS. These results indicate that CSCs-exosomes have a notable ability to induce proliferation, metastasis, and angiogenesis, leading to a remarkable tumor growth and progression. Consistently, a previous study showed the ability of exosomes derived from CD90^+^ CSCs to induce angiogenesis in HCC through a similar molecular mechanism [29]. CSC-derived exosomes can also reprogram AD-MSCs into myofibroblast-like cells, which subsequently maintain tumor growth and angiogenesis [30]. Moreover, the uptake of HCC-derived exosomes induces migration and invasive abilities of nonmotile normal liver cells through activation of PI3K/AKT and mitogen-activated protein kinase (MAPK) signaling pathways and their downstream targets MMP2 and MMP9 [45].

Interestingly, exosomes from either CSCs or MSCs have no significant effect on activities of antioxidant enzymes (SOD, GPX, and CAT) or level of lipid peroxidation marker MDA in HCC liver as compared to the PBS group, which exhibited lower enzymatic activities and higher MDA levels. This suggests that oxidative stress/antioxidant pathway may not be involved in stimulatory or inhibitory effects of CSCs- or MSCs-exosomes, respectively, on HCC progression. In contrast to our results, Dutta et al. [46] found that exosomes derived from breast cancer cell lines can induce oxidative stress damage to the recipient human primary mammary epithelial cells. These contradictory results may be attributed to variation in cancer type and the target cells analyzed. Dutta et al. [46] stimulated normal mammary epithelial cells with tumor exosomes. This treatment enhanced oxidative stress response in normal cells. On the contrary, we focused on hepatic cells already transformed, derived from rats with big foci of HCC cells in the liver. These cells have been already modified and prone to show high levels of oxidative stress as compared to normal cells (PBS group versus normal group).

Mobilization of residential MSCs into tumors may indicate a significant role for MSCs in tumors. Researchers have used some tumor models in which exogenous MSCs and their derived exosomes are administrated to investigate their effect on tumor progression. Interestingly, several studies revealed contradicting results, with some researchers finding that MSCs/MSCs-exosomes induce tumor development and others reporting a tumor-suppressive effect. Our results agree with Bruno et al. [23] and Ko et al. [24] who reported that MSCs-exosomes induced tumor regression when injected either locally in HepG2 cell-induced subcutaneous tumor in nude mice or intravenously in rat N1S1 cell-induced hepatic tumor, respectively. In contrast, Zhu et al. [47] found that MSCs-exosomes induced growth of subcutaneous tumor, formed by HCC cell line xenograft, in nude mice. The reason for this discrepancy is unknown, but it may be attributed to the timing of exosome injection either before or after tumor formation [48]. In the present study and experiments of Bruno et al. [23] and Ko et al. [24], exosomes were administrated after tumor initiation, while Zhu et al. [47] coinjected tumor cells with exosomes; however, this needs further investigation. A similar contradiction was reported for MSCs that induced tumor progression when coadministrated with tumor cells [49], but induced tumor regression when injected into established tumors [50, 51]. Thus, it is likely that the presence of MSCs or their exosomes in the microenvironment of tumor during the initial stage could enhance angiogenesis that is required for tumor onset or create tumor stem cell niche [47, 49]. However, when they were injected in established tumors, they inhibited angiogenesis and induced apoptosis, leading to tumor regression [23, 50, 51]. In support, we also found a higher apoptotic rate (indicated by increased expression of apoptotic genes, *Bax* and *p53*, decreased expression of the antiapoptotic gene, *Bcl2*, and weak immunostaining of the nuclear proliferative marker PCNA) and decreased angiogenetic activity (revealed by downregulation of *VEGF* gene) following injection of BM-MSC-derived exosomes in established HCC. Activation of the gatekeeper of the genome p53 by MSCs-exosomes may enhance the tumor suppression functions, a fundamental inhibitory mechanism of oncogenesis. Another possible explanation for controversial results of MSCs-exosomes on tumor growth may be the heterogeneity of these exosomes, as their donors (MSCs) coming from different sources so the produced exosomes may also carry different molecules which may promote or repress tumor development. In addition, the different tumor types, *in vivo* tumor models, and the variation in the way and time of exosome administration may also influence the effect of exosomes on tumor progression [48].

Exosomes mediate cell-to-cell communication through the transfer of their cargoes of proteins, mRNAs, miRs, and lncRNAs [52] that participate in the genetic exchange among cells [53]. To provide valuable insight into the underlying molecular mechanism of exosomes, we detected the expression of some miRs and lncRNAs present in the HCC microenvironment. Administration of CSCs-exosomes increased the expression of HCC exosomal miR21 and decreased the expression of liver miR122 and HCC miRs (miR148a, miR16, and miR125b) than that of HCC liver injected by PBS. This expression profile for miRs was reversed in the MSC-Ex group. Among these miRs, miR122 is a liver-specific miR constituting 70% of the liver miRs [54] and its downregulation associated with HCC progression in humans and rodents [55, 56]. However, miR21, as a liver-specific antiproliferative miR, is overexpressed in HCC cells [57] and promotes their proliferation and metastasis [58]. Consequently, miR21 inhibition induces apoptosis in CSCs [59]. In addition, miR122-loaded MSC exosomes increased the chemosensitivity of HCC cells both *in vitro* and *in vivo* [10], indicating a potential role for MSCs-exosome, through their antitumor miRs, in HCC treatment. In consistence, we also found increased expression of miR122 along with increased apoptosis in HCC liver following injection of MSCs-exosomes. The anti-HCC effect of miR122 is prevented by IGF1 produced by HCC cells to ensure their own proliferation [32]. Thus, in the present study, CSCs may induce HCC progression through downregulation of miR122 in the tumor microenvironment, but this needs experiments using mimic122 and anti-miR122 to confirm this hypothesis. Similarly, anti-HCC miR148a and miR125b exert inhibitory effects on epithelial mesenchymal transition (EMT) and CSC-like phenotypes by targeting TGF*β*1, which is a potent stimulator for EMT in the tumor microenvironment and induces the transformation of liver stem cells into CSCs in HCC [60]. In agreement, animals injected by CSCs-exosomes exhibited a decreased level of these two miRs (miR148a and miR125b) and an increased serum level of TGF*β*1 and hepatic expression of TGF*β*1 mRNA and protein. miR148a is also a proapoptotic miRNA which represses *Bcl2* [61]. This may explain decreased apoptosis in HCC cells of the CSCs-Ex group which had lowest miR148a and highest *Bcl2* mRNA levels. In contrast, MSCs-exosomes' repressive role on HCC may be mediated by high miR148a and low Bcl2 mRNA and TGF*β*1 mRNA and protein levels. Furthermore, MSCs-exosomes are highly enriched in antiangiogenic miR16 that suppresses *VEGF* expression thereby favoring the inhibition of angiogenesis in recipient breast cancer cells (reviewed by [62]). In a similar way, we found upregulation of miR16 and *VEGF* gene in livers of HCC rats receiving BM-MSCs.

lncRNAs play an important role in acquisition of CSC phenotype, and so their targeting may be a novel therapeutic strategy for HCC. Indeed, recent studies reported that targeted inhibition of lncDANCR decreases tumor development in subcutaneous and intrahepatic tumor models of mice [63]. In agreement, we also found that intrahepatic injection of CSCs-exosomes led to a significant increase in the exosomal lncTuc339, lncHEIH, and the HCC lncHOTAIR. Again, the expression of these three lncRNAs was downregulated in livers of the MSC-Ex group. In consistence with our results, a recent study by [15] showed a stimulatory effect for the most highly expressed lncRNA in HCC cell-derived exosomes, TUC339, on HCC cell proliferation and metastatic potential due to decreased cellular adhesion in ECM. Similarly, lncHOTAIR promotes neoplastic transformation of normal liver stem cells into CSCs, leading to progressive metastasis and enhanced tumorigenic potential mainly through induction of EMT [64]. lncRNA-HEIH expression in serum and exosomes was also significantly increased in patients with HCV-related HCC, indicating the positive role for this lnc in HCC progression [12]. Although targeting miRs and lncRNAs may be a promising novel therapeutic approach for HCC, and other cancers, many puzzles remain to be solved before clinical trials. Targeting miRs and lncRNAs may not only kill HCC cells but also kill normal cells through inhibition of essential signal pathways regulated by these miRs and lncRNAs. Thus, a precise understanding the properties of miRs and lncRNAs in the HCC microenvironment will aid in selective targeting of these RNA molecules which will be a promising approach for liver cancer treatments.

Despite their ability to restore liver homeostasis through repair and regeneration of hepatocytes and their successful use as a potential therapeutic approach in different *in vitro* and *in vivo* models of HCC, MSC-exosome-mediated therapy faces some obstacles. The practical limitation for large-scale MSC-exosome production and for their isolation from MSC media without modification of their cargoes is among the most common obstacles. Exosome heterogeneity (similar to heterogeneity of their source, MSCs) is another hurdle which may lead to different actions on their recipient cells. Therefore, before application of exosome-mediated therapy in patients, effective methods that keep the homogeneity of MSC-derived exosomes need to be developed. In general, using MSCs-exosomes in HCC treatment should be performed with caution because their role in tumor development has not completely elucidated yet. Identification of the underlying mechanism involved in the modulation of MSC-derived exosomes is pivotal to determine their actual role in tumor development and guide researchers in the development of effective treatment through their precise modification.

## 5. Conclusion

The obtained data demonstrate that CSCs- and MSCs-exosomes remarkably induce and inhibit, respectively, tumor development and progression *in vivo*. To the best of our knowledge, this is the first study to report the effect of CSCs-exosomes and MSCs-exosomes on a rat long-term model of HCC induced by DEN. The exosome-mediated interactions between CSCs/MSCs and tumor cells may modulate signaling pathways related to apoptosis, metastasis, and angiogenesis in tumor cells. Although our results provide valuable insight into the effect of CSCs- and MSCs-exosomes on tumor growth and progression *in vivo*, further investigations will be needed to elucidate how different exosomes in the tumor microenvironment react together and how they can be reliably targeted in patients with HCC.

## Figures and Tables

**Table 1:** Primers used for real-time PCR.

Gene	Forward primer	Reverse primer
*p53*	GTTCCGAGAGCTGAATGAGG	TTTTATGGCGGGACGTAGAC
*Bax*	ACACCTGAGCTGACCTTG	AGCCCATGATGGTTCTGATC
*Bcl2*	AGTACCTGAACCGGCATCTG	CATGCTGGGGCCATATAGTT
*TGFβ1*	AAGAAGTCACCCGCGTGCTA	TGTGTGATGTCTTTGGTTTTGTCA
*NFκβ*	CCTAGCTTTCTCTGAACTGCAAA	GGGTCAGAGGCCAATAGAGA
*VEGF*	GATCATGCGGATCAAACCTCACC	CCTCCGGACCCAAAGTGCTC
*MMP9*	TCGAAGGCGACCTCAAGTG	TTCGGTGTAGCTTTGGATCCA
*TIMP1*	CGCAGCGAGGAGGTTTCTCAT	GGCAGTGATGTGCAAATTTCC
*β-Actin*	AAGTCCCTCACCCTCCCAAAAG	AAGCAATGCTGTCACCTTCCC

## Data Availability

The data used to support the findings of this study are available from the corresponding author upon request.

## Abbreviations

AD: Adipose
BM: Bone marrow
CSCs: Cancer stem cells
DEN: Diethylnitrosamine
Ex: Exosome
HCC: Hepatocellular carcinoma
lncRNA: Long noncoding RNA
miR: Micro RNA
MSCs: Mesenchymal stem cells.
